# Current Status of α-Synuclein Biomarkers and the Need for α-Synuclein PET Tracers

**DOI:** 10.3390/cells14161272

**Published:** 2025-08-18

**Authors:** Sara E. Berman, Andrew D. Siderowf

**Affiliations:** Parkinson’s Disease and Movement Disorders Center, Department of Neurology, Pennsylvania Hospital, Perelman School of Medicine, University of Pennsylvania, Philadelphia, PA 19104, USA

**Keywords:** Parkinson’s disease, dementia with Lewy Bodies, α-synuclein

## Abstract

Synucleinopathies are neurodegenerative disorders defined by the pathological aggregation of α-synuclein. Several α-synuclein biomarkers have been developed to aid diagnosis and research, such as cerebrospinal fluid (CSF) and blood-based measurements, seed amplification assays (SAAs), and immunohistochemical detection from skin biopsies. While these existing biomarkers have important uses, they face limitations in diagnostic specificity, spatial localization, and the ability to monitor disease progression or response to therapy. The development of α-synuclein PET tracers, which would allow for the direct in vivo imaging of α-synuclein, represents an important unmet need in both research and the clinical care of patients with movement disorders. This review outlines the current landscape of α-synuclein biomarkers and discusses both the scientific and technical challenges in developing α-synuclein PET imaging tracers.

## 1. The Urgent Need for α-Synuclein Imaging

Synucleinopathies are a group of neurodegenerative diseases defined by the aberrant aggregation of α-synuclein protein in neurons and glial cells. Clinical synucleinopathies include Parkinson’s disease (PD), dementia with Lewy bodies (DLB), and multiple system atrophy (MSA) [1]. While the normal function of α-synuclein is not well understood, it is postulated to be involved in synaptic vesicle regulation [2]. When α-synuclein aggregates abnormally, it forms insoluble fibrils that assemble into intracellular inclusions, including Lewy Bodies [3] and Glial Cytoplasmic Inclusions [4], which are the pathologic hallmarks of this family of disorders. Insoluble α-synuclein aggregates are thought to contribute to cellular dysfunction and neurodegeneration, possibly by interfering with protein degradation pathways or activating neuro-inflammatory cascades [5,6]. Currently, there are no effective disease-modifying therapies for synucleinopathies and the available treatments are limited to symptomatic management [7]. As seen in Alzheimer’s disease (AD), with the advent of amyloid PET imaging [8,9], the development of molecular imaging techniques that allow for the in vivo visualization of α-synuclein could greatly enhance research efforts and serve as a valuable adjunct in clinical care by enabling earlier diagnosis and more precise monitoring of disease progression in patients with syuncleinopathies and other Parkinsonian syndromes.

## 2. Synucleinopathies: Clinical and Biological Spectrum

PD is the most common and well-recognized synucleinopathy [10]. Syunclein pathology, consisting primarily of Lewy bodies, which are cytoplasmic inclusions, and Lewy neurites, affects dopaminergic neurons in the substantia nigra, leading to motor symptoms such as tremor, rigidity, postural instability, and bradykinesia. [1,3]. However, synuclein pathology in PD is widespread, affecting both the central and peripheral nervous system [11,12]. This extensive, spatially expansive pathology gives rise to a spectrum of non-motor symptoms, including autonomic dysfunction with constipation and blood pressure dysregulation, as well as neuro-behavioral features such as anxiety, depression, cognitive impairment, and hallucinations [13,14].

DLB is another major synucleinopathy characterized by the presence of Lewy pathology in cortical and limbic structures [15,16]. This distribution of pathology is associated with progressive cognitive impairment and ultimately dementia. DLB shares pathological features with PD, including α-synuclein accumulation int Lewy bodies and neurites which are morphologically and biochemically indistinguishable from those in PD [17]. While both diseases involve Lewy body pathology, DLB typically manifests with earlier cognitive decline, including memory impairment, executive dysfunction, and more prominent visual hallucinations and alterations in arousal compared to PD [18,19]. The overlap in symptoms and pathology between PD and DLB complicates diagnosis but advances in neuroimaging and biomarkers may provide clearer diagnostic tools in the future [20].

Rapid eye movement (REM) sleep behavior disorder (RBD) is a parasomnia that often has underlying α-synuclein pathology. RBD involves the loss of normal muscle atonia during REM sleep, leading to the enactment of vivid and frequently violent dreams. This disorder is a well-established prodromal marker of neurodegenerative diseases, including PD and DLB, and is thought to result from α-synuclein-related degeneration in brainstem regions that regulate sleep [21,22]. Studies have shown that RBD can precede the onset of motor symptoms by years, providing an early indicator or predictor of synucleinopathies [21,23].

MSA is another synucleinopathy, but differs from PD and DLB in both its clinical presentation and pathological underpinnings. MSA involves widespread neurodegeneration that affects both the central and peripheral nervous systems, leading to symptoms such as autonomic dysfunction, cerebellar ataxia, and parkinsonism. MSA is characterized by the accumulation of α-synuclein in oligodendrocytes, distinguishing it from the predominantly neuronal involvement of PD and DLB [24,25]. The distinct clinical and pathological features of MSA compared to PD and DLB highlight the heterogeneity within synucleinopathies. As with PD and DLB, MSA may be preceded by symptomatic RBD. However, MSA is associated with more rapid progression and reduced life expectancy, particularly relative to PD [26].

From a biochemical perspective, there has been important work identifying different strains of α-synuclein involved in the syuncleinopathies. The different strains (or conformations) of α-synuclein may have varying degrees of tropism for different cell types, e.g., neurons vs. glial cells [27]. Using CSF samples from patients with PD and MSA, Shahnawaz and colleagues demonstrated that α-synuclein fibrils from PD patients had a larger twisting distance than those obtained from patients with MSA—the fibrils were structurally distinct across different disease subtypes [28].

The pathologic hallmark of synucleinopathies (α-synuclein aggregation) and different syunclein strains associated with different disease phenotypes have led to ongoing research into biomarkers for early diagnosis and potential therapeutic strategies, and will be the focus of this review.

## 3. Need for Biomarkers

There is a pressing need for reliable biomarkers that can be used both in the context of clinical care and as research tools for synucleinopathies (Table 1). From the standpoint of clinical care, one of the primary goals is to be able to offer patients a diagnosis that is not only accurate but also made as early in the disease process as possible. At present, the gold-standard method for diagnosing PD continues to be a detailed clinical evaluation. In particular, neurologists who have received specialized fellowship training in movement disorders are generally highly accurate in making these diagnoses, as demonstrated in recent studies, with greater accuracy than clinicians without subspeciality training in movement disorders [29]. Nonetheless, expert clinical assessments can be prone to error, especially when it comes to identifying early presentations of atypical parkinsonian syndromes [30,31]. As such, there remains room for improvement through the incorporation of objective biomarker tools into routine diagnostic workflows.

In addition to improving diagnostic accuracy, the availability of biomarker-based testing could provide a valuable option for patients who desire a diagnosis grounded in molecular or biological evidence, rather than one that is solely dependent on a physical examination. This may be especially appealing to patients seeking clarity or validation through measurable, lab-based data.

Single-photon emission computerized tomography (SPECT) imaging of the dopamine transporter (DAT-SPECT) is an imaging modality that is currently used in clinical practice. I-123 Ioflupane imaging has been shown to be sensitive and specific in detecting loss of dopamine integrity in patients with clinical signs or parkinsonism or tremor [32,33] and can detect abnormalities on the unaffected side in patients with early, unilateral PD [34]. While useful, DAT-SPECT imaging has notable limitations. For instance, a positive DAT scan, which indicates reduced dopaminergic activity, may also be observed in several non-dopaminergic or non-Parkinson’s-related conditions, such as progressive supranuclear palsy (PSP), corticobasal degeneration (CBD), and normal-pressure hydrocephalus (NPH) [35,36]. Furthermore, certain medications that also bind to the dopamine transporter—such as bupropion, amphetamines, benztropine, and cocaine—can also interfere with the results, leading to false positives or equivocal interpretations [37,38]. These factors complicate the utility of DAT scans as definitive diagnostic tools, especially in patients with complex medical or psychiatric backgrounds for whom medication holidays (e.g., of bupropion) cannot be performed.

In the research setting, the need for accurate, specific, and sensitive biomarkers is equally critical. Such tools are essential to ensure the correct identification and enrollment of participants in clinical trials, particularly those testing disease-modifying therapies that are targeted at specific pathological processes [39]. The importance of precise participant selection is underscored by the fact that in the earliest stages of parkinsonian disorders, clinical symptoms can be subtle and overlapping, making it difficult to distinguish between PD and its atypical variants [40]. Therefore, the successful development of robust biomarkers would significantly enhance both clinical decision-making and the efficiency of clinical trials, ultimately leading to improved outcomes for patients.

## 4. Status of Currently Available α-Synuclein Biomarkers

### 4.1. Quantitative Measurement of α-Synuclein in Cerebrospinal Fluid

Cerebrospinal fluid (CSF) α-synuclein has emerged as a promising biomarker for PD. The accumulation of aggregated α-synuclein in the brain is a key feature in the progression of the disease, leading to neurodegeneration. Measuring α-synuclein levels in the CSF offers a potential non-invasive way to assess the presence and progression of PD [41].

One of the difficulties in the quantitative measurement of α-synuclein is that it exists in multiple forms, including monomers, oligomers, and fibrils, which are the building blocks of the larger aggregates [42]. In addition, α-synuclein may exist in a phosphorylated or unphosphorylated state. As aggregated and hyperphosphorylated α-synuclein comprise Lewy Bodies [3], researchers have sought to develop antibodies that are able to bind to and subsequently measure this conformation of α-synuclein [43]. Additionally, researchers have also sought to measure oligomeric α-synuclein, as oligomeric α-synuclein has been proposed as the toxic form of alpha synuclein in neurodegenerative diseases [43,44].

CSF α-synuclein levels have been found to be altered in patients with PD compared to healthy controls, though the results are inconsistent across studies and the results vary depending on which α-synuclein species is measured. Some studies report decreased CSF α-synuclein levels in PD patients, possibly due to sequestration in Lewy bodies or other forms of cellular dysfunction [45,46,47]. In contrast, as reviewed by Gao and colleagues, other research suggests that α-synuclein levels are elevated in the CSF of PD patients [48]. The variation in results may be attributed to differences in disease stage, sample handling, and the analytical techniques used. Moreover, the aggregation state of α-synuclein, such as whether it is in its monomeric, oligomeric, or fibril form, or the strain, may influence the biomarker’s sensitivity and specificity in detecting PD [49]. In addition, studies looking at oligomeric α-synuclein and phosphorylated α-synuclein have shown initial promise but consistent replication has not been reported.

Beyond diagnosing PD, CSF α-synuclein is also being explored as a potential biomarker for monitoring disease progression and therapeutic efficacy. Its levels could provide insights into the dynamics of protein aggregation over time, offering a valuable tool for assessing disease status in clinical trials or routine clinical practice [50]. However, further research is needed to standardize the protocols for measuring CSF α-synuclein and to determine its exact role in the early detection and prognosis of PD. Additionally, measurements from CSF or serum homogenate samples do not allow for an understanding of the spatial distribution of pathology, which can potentially be addressed using other synuclein biomarker methods, as discussed below.

### 4.2. CSF α-Synuclein Seed Amplification Assay

CSF α-synuclein levels alone have shown limited success in differentiating between various synucleinopathies, including DLB, MSA, and PD [48]. A notable advance in α-synuclein biomarker research is the development of the α-synuclein seed amplification assay [28,51,52]. This technique involves adding recombinant monomeric α-synuclein to a CSF sample, which may or may not contain pathologic α-synuclein (known as “seeds”). If these seeds are present, they recruit the monomers, leading to the formation of fibrils. These fibrils are then labeled and measured using the fluorescent dye thioflavin-T (ThT) [53]. The α-synuclein seed amplification assay was studied in 1123 participants in the Parkinson’s Progression Markers Initiative, showing excellent diagnostic performance in distinguishing PD from healthy controls, with 87.7% sensitivity for PD and 96.3% specificity for healthy controls [54]. Additionally, in a study involving 129 CSF samples from patients in Scandinavia, the α-synuclein seed amplification assay successfully distinguished between synucleinopathies (such as PD and MSA) and tauopathies (such as CBD and PSP) [55]. In order to address the concern of reproducibility across laboratories, Russo and colleagues tested PPMI samples in syunclein seeding assays in three different laboratories, finding high sensitivity (86–96%) and sensitivity (93–100%) [56]. There are some limitations of the seed amplification assay, however, that are important to address; ThT fluorescence is sensitive to high concentrations of α-synuclein fibrils [57], which limits its sensitivity to preclinical disease states.

### 4.3. Assays to Measure α-Synuclein in Blood

In addition to CSF, there has been an effort to develop assays to assess the presence of α-synuclein in blood, specifically in serum and/or plasma samples. Researchers have been able to measure α-synuclein in neuronally derived extracellular vessels (EVs) found in the plasma of patients with PD [58,59]. This requires the purification of neuronal-derived extracellular vesicles using an NCAM-1 (anti-neuronal cell adhesion molecule) antibody prior to use of the seeding assay [58]. There has been some controversy, however, regarding the use of this EV method for the measurement of α-synuclein and its reproducibility [60].

Using immunoprecipitation in conjunction with real-time quaking-induced conversion (IP/RT-QuIC) Okuzumi and colleagues reported the ability to measure α-synuclein directly from the serum in patients with PD compared to normal controls [61]. Additionally, they were able to identify different confirmations (strains) of synuclein seeds in different syuncleinopathies—paired filaments or multiple bundled filaments in serum from patients with PD and DLB, and twisted filaments and straight filaments in serum from MSA patients [61].While promising, these results need to be replicated in independent cohorts and in other laboratories.

There has also been some burgeoning work to identify α-synuclein in other body fluids, including saliva. [62,63,64]. In a study by Angius and colleagues, although total α-synuclein did not differ between the saliva of PD patients and healthy controls, the ratios of different subtypes of α-synuclein in saliva show some promise [63].

### 4.4. Florescent Immunohistochemistry of α-Synuclein from Skin Biopsies

Another approach to measuring α-synuclein pathology is synuclein skin biopsy. In initial reports, skin biopsies appear to give similar accuracy compared to spinal fluid seed amplification assays in patients with PD [65]. This screening method involves taking three skin punch biopsies at disparate locations: the distal leg 10 cm above the lateral malleolus, the distal thigh 10 cm above the lateral knee, and the posterior cervical region 3 cm lateral to the C-7 spinous process [65]. Importantly, the location of α-synuclein deposits in the skin is key for distinguishing MSA from PD. In MSA, abnormal α-synuclein deposits are predominantly found in the somatic regions of the skin, particularly in the lower limbs, while in PD, these deposits are more commonly detected in the autonomic regions [66]. This difference in deposit distribution helps differentiate MSA from PD, offering valuable insights for diagnosis; however, this needs further confirmation in additional research and patient samples [67].

Syunclein skin biopsies, although newly developed, are already demonstrating importance in advancing clinical care. In a study of 97 patients with suspected synucleinopathy (54 with PD; 19 with DLB), following skin biopsy, 66% of patients had a change in diagnosis and 55% had a change in treatment [68]. An example of a change in diagnosis was in patients with a prominent action tremor, initially with suspected ET, in which diagnosis was changed to a parkinsonian syndrome after a positive synuclein skin biopsy. With respect to change in treatment, in patients with parkinsonism and inadequate levodopa response, a negative synuclein skin biopsy was suggestive of an alternative diagnosis (e.g., PSP) and levodopa was stopped [68].

## 5. Overview of Imaging Biomarker Approaches

Forms of multimodal biomarker imaging, including structural imaging, FDG-PET, and disease protein-specific nuclear medicine imaging, have greatly increased our ability to understand neurodegenerative diseases, both from research and clinical perspectives.

### 5.1. Structural Imaging

Structural imaging has been explored for the differential diagnosis of PD and for measuring progression. Imaging the substantia nigra can detect abnormal signal, even in the early stages of disease. Volumetric analyses of cortical and subcortical regions have demonstrated significant atrophy in the putamen and parietal cortex, correlating with disease progression. In addition, MRI-based multivariate gray matter volumetric assessments show promise in predicting the progression of motor symptoms [69,70]. There have also been recent advances in utilizing machine learning techniques to harness structural imaging in the study of syuncleinopathies. Specific brain regions, including the substantia nigra, putamen, and other basal ganglia structures were examined for changes associated with different parkinsonian syndromes and machine learning models were trained to recognize patterns in the brain’s anatomical features, differentiating the neuroanatomical signatures of PD, MSA-P, and PSP with high sensitivity and specificity [71].

### 5.2. FDG-PET

Eidelberg and colleagues used FDG-PET to understand the topographic organization of parkinsonism, which has since been termed the Parkinson’s Disease-Related Covariance Pattern [72]. The PDRP was associated with increased metabolic activity in the lentiform nucleus and thalamus and decreased activity in the lateral frontal, paracentral, inferior parietal and parieto-occipital areas in individuals with PD [72]. In a subsequent study in 2007, the PDRP was validated in patients with PD and healthy volunteers, and found to reliably and reproducibly demonstrate the aforementioned regional metabolic abnormalities in the PD cohort [73].

### 5.3. Amyloid and Tau Imaging

With the advent of anti-amyloid therapies, amyloid PET is beginning to enter increasing clinical use and lessons learned from amyloid research may inform the development of synuclein tracers. Currently available amyloid tracers are either carbon-11-based (PIB) or F-18-based (florbetapir, florbetapen and flutematol) [8]; however, only F-18 tracers are relevant to clinical practice. The use of amyloid PET imaging in research focusing on the AD continuum is widespread, having been used in studies to predict cognitive trajectories as well as those on the conversion from MCI to dementia [74,75]. Clinically, amyloid PET is now being used to determine who may be a candidate for anti-amyloid infusion therapies and to clarify dementia etiologies in confusing clinical cases [8].

In addition to amyloid-PET imaging, there have also been advances in the molecular imaging of tau. Tau-PET imaging (combined with CSF tau biomarkers in certain studies) has been able to predict future cognitive decline in cognitively unimpaired individuals, which could potentially increase screening efficiency in early-stage clinical trials for AD [76,77]. Currently available tau-PET tracers are specific for tau in AD neurofibrillary tangles [78]. The history of tau imaging development illustrates the difficulties of tracer development—specifically addressing problems with off-target binding and the multiple isoforms of tau [79,80,81].

## 6. Molecular Imaging in Synucleinopathies and the Progression of α-Synuclein Specific Imaging

Because of the importance of monitoring α-synuclein pathology, both for clinics and in research, and the limitations of available biomarkers, there is an urgent need for α-synuclein imaging tracers. A number of research teams are actively pursuing this goal (Table 2). However, the development of α-synuclein PET tracers faces several significant challenges. One key issue is achieving adequate binding affinity to the appropriate α-synuclein species. Other issues are the pharmacokinetic properties that make a tracer feasible in the clinic and the minimization of off-target binding, particularly to other misfolded proteins, such as amyloid-beta and tau [82,83]. These difficulties underscore the urgent need for better tracers and diagnostic tools to track the progression and treatment of α-synucleinopathies [84]. The next section will briefly summarize the current state of different α-synuclein PET tracers.

Endo and colleagues have reported pre-clinical and preliminary clinical findings for their tracer, [F18]-C05-05. They report excellent affinity based on studies of homogenized brain tissue from patients with PD, DLB, and MSA [85]. Clinical studies showed the binding of [F18]-C05-05 in the mid-brain and striatum of patients with PD, DLB, and MSA. However, the authors also found evidence for binding to amyloid-beta and tau, indicating that the compound may not be specific to a-synuclein pathology. Nonetheless, the strong affinity of this tracer shows the potential to image α-synuclein in vivo with PET, and further refinement of this molecule may be possible.

**Table 2:** Summary of existing α-synuclein biomarkers.

Name	Modality	Authors
***Clinically Available***		
Alphα-synuclein Skin Biopsy	Skin biopsy, immunohistochemistry	Gibbons et al., 2023 [67]; Gibbons et al., 2024 [65]
Alpha Synuclein Seed Amplification Assay	Seed amplification assay, CSF	Concha-Marambio et al., 2023 [53]; Fernandes Gomes et al., 2023 [55]

***In Development***		
**PET tracers**		
[F18]ACI-12589 PET	PET-CT	Smith et al., 2023 [86]
[F8]-SPAL-T-06	PET-CT	Matsuoka et al., 2022 [87]
[F18]-F0502B	PET-CT	Xiang et al., 2023 [88]
[F18]-C05-05	PET-CT	Endo et al., 2024 [85]
[3H] and [11C] KAC-50.1	PET-CT	Saturino Guarino et al., 2024 [89]

**Fluid-based markers**		
Alpha Syunclein Seed Amplification Assay	Seed amplification assay, serum	Okuzumi et al., 2023 [61]
Alpha-Syunclein Seed Amplification Assay	Isolation of extracellular vesicles using NCAM-1, followed by seed amplification assay, plasma	Bernhardt et al., 2024 [60]

Using an [F18]ACI-12589 PET ligand, Smith and colleagues studied 23 individuals with α-synuclein-related disorders, 11 participants with other neurodegenerative disorders, and 8 control subjects. The [F18]ACI-12589 PET ligand showed high uptake in the white matter and cerebellar peduncles of the participants with MSA; however, this was not seen in participants with PD or DLB [86]. This is in contrast to [F18]-C05-05, which bound effectively to α-synuclein in PD and DLB [85]. Regarding another F18-PET ligand—in three patients with MSA—the fluorinated PET ligand [F8]-SPAL-T-06 was able to bind to α-synuclein in the putamen, pons, and cerebellar peduncles [87].

Xiang and colleagues developed [F18]-F0502B, which has high binding affinity for α-synuclein, but not amyloid or tau [88]. Autoradiography in human brain samples from patients with PD and AD found that [F18]-F0502B bound to Lewy bodies in the PD brains, with negligible biding in AD brains. Studies using this compound are ongoing. In addition, studies with other tracers that are under development are discussed in contributions to this issue.

## 7. Conclusions

Positron emission tomography (PET) imaging of α-synuclein is emerging as a highly promising and dynamic area of investigation, both for research and to improve the diagnosis and management of individuals living with synucleinopathies. Looking ahead, there is a critical need to develop tracers that demonstrate enhanced binding affinity, allowing for the detection of small quantities of α-synuclein. Another important objective is the design of radiotracers that can selectively identify whether α-synuclein is localized within neurons or glial cells, as this distinction may provide important insights into disease progression and phenotype; the PET tracers currently under development do not appear to have this degree of resolution, nor are they specific to syunclein strain type. In parallel with this, antibody- and aptamer-based imaging strategies are being explored as complementary approaches, with the potential for the highly selective targeting of specific α-synuclein conformations, strains, or cell-type localizations [90,91]. Progress in these areas would not only advance the field’s understanding of the complex biological processes underlying parkinsonian syndromes but may allow for earlier, pathology-based diagnosis and staging based on the extent of synuclein pathology in a manner that echoes staging at autopsy [11]. Ultimately, these innovations could lead to a paradigm shift in how both researchers and clinicians approach the study and treatment of synuclein-related neurodegenerative disorders.

## Figures and Tables

**Table 1:** Unmet needs in α-synuclein biomarkers.

Unmet Need	Potential Biomarker(s)
Differentiation between clinico-pathologic disorders	Seed amplification assay; α-synuclein skin biopsy; spatial distribution of PET tracers (e.g., [F18]ACI-12589 in MSA)
Monitoring disease progression	Quantitative fluid biomarkers in plasma and/or CSF; PET-CT imaging
Tracking response to therapies	Quantitative fluid biomarkers in plasma and/or CSF; PET-CT imaging

## Data Availability

No new data were created for this review article.

## Abbreviations

The following abbreviations are used in this manuscript:

PD	Parkinson’s disease
RBD	REM Sleep Behavior Disorder
MSA	Multiple Systems Atrophy
AD	Alzheimer’s disease
REM	Rapid Eye Movement
